# Mobile-bearing versus Medial-pivot Designs in Total Knee Arthroplasty: A Meta-analysis

**DOI:** 10.1186/s43019-025-00280-7

**Published:** 2025-07-24

**Authors:** Huu Dat Nguyen, Le Hoan Nguyen, Nguyen Anh Duy Tran, Quang Son Tran, Tu Thai Bao Nguyen, Thanh Tan Nguyen

**Affiliations:** 1 Department of Orthopedics, Faculty of Medicine, Can Tho University of Medicine and Pharmacy, 179 Nguyen Van Cu Street, Tan An Ward, Can Tho City, Vietnam; 2 Department of Orthopedics and Neurosurgery, Can Tho University of Medicine and Pharmacy Hospital, Can Tho University of Medicine and Pharmacy, Can Tho, Vietnam

**Keywords:** Knee arthroplasty, Mobile-bearing, Medial-pivot, Outcomes, Meta-analysis

## Abstract

**Background:**

Mobile-bearing (MB) and medial-pivot (MP) prostheses are the two popular designs used in total knee arthroplasty (TKA), yet their long-term outcomes remain controversial. This meta-analysis was conducted to determine whether there are any differences in outcomes between patients who receive these two prostheses in TKA.

**Method:**

We comprehensively searched electronic databases up to November 2024. Observational studies or randomized controlled trials reported postoperative outcomes, including radiographic results, functional score through the Knee Society Score (KSS), Western Ontario and McMaster Universities (WOMAC) score, and patient satisfaction. The results are presented in odd ratios (ORs) or mean differences with corresponding 95% confidence intervals (CIs).

**Results:**

In total, four studies were included, with 1069 patients and 1251 operated knees (632 MB TKAs and 619 MP TKAs). The mean age was 68.21 years old and the mean follow-up duration was from 5 to 11 years. Regarding patient satisfaction, the MB prostheses brought greater comfort to patients in the last follow-up than the MP group (OR = 3.19; 95%CI 1.92–5.30; *p* < 0.001). Additionally, there were no differences in the outcomes of functional scores, including the KSS (*p* = 0.34), WOMAC score (*p* = 0.16), and range of motion (ROM) (*p* = 0.36) between patients receiving the MB and MP prostheses.

**Conclusions:**

Our meta-analysis of four studies highlighted the long-term outcomes of TKA between MB and MP designs. While KSS, WOMAC scores, and knee ROM showed no significant differences, patients with MB designs reported higher satisfaction levels compared with those with MP designs.

**Supplementary Information:**

The online version contains supplementary material available at 10.1186/s43019-025-00280-7.

## Introduction

Total knee arthroplasty (TKA) has emerged as a transformative solution for managing knee osteoarthritis, offering pain relief, restoring function, and improving quality of life [1–4]. In the decades since the first fixed-bearing design, innovations in prosthetic design have rapidly evolved to enhance functional performance and minimize long-term complications. Based on the bearing surface design, mobile-bearing (MB) and medial-pivot (MP) designs have emerged as two widely used options due to their distinct biomechanical principles [3, 5–8].

The MB design aims to enhance tibiofemoral congruency, reduce wear, and improve range of motion (ROM) by allowing anterior–posterior insert movement [2, 9, 10]. However, concerns have been raised regarding its long-term functional outcomes and impact on patient quality of life, particularly related to knee kinematics owing to the anterior translation of the tibial insert [11–14]. In contrast, the MP design seeks to replicate natural knee kinematics by stabilizing the medial compartment while permitting controlled lateral movement, potentially enhancing joint stability and patient satisfaction [6, 15–17].

Despite their theoretical advantages, clinical comparisons between MB and MP designs remain inconclusive. Some studies report better satisfaction and fewer complications with MB implants [5, 14], while others find comparable or even superior functional outcomes for MP designs [7, 18]. In addition, these discrepancies in the literature highlight the need to identify the potential advantages and limitations of each design to guide clinical practice in TKA, which may ultimately lead to different outcomes in patients.

This meta-analysis aims to synthesize existing evidence to provide a clearer understanding of the functional outcomes and patient satisfaction associated with MB and MP designs in TKA. By addressing inconsistencies in the literature, we seek to inform clinical decision-making and guide future research on prosthetic design selection.

## Methods

### Registration and protocol

This research adhered to the guidelines set forth by the Preferred Reporting Items for Systematic Reviews and Meta-Analyses. Additionally, it has been registered with the International Prospective Register of Systematic Reviews under the registry ID CRD42024621090.

### Data sources and search strategies

Two independent reviewers conducted an extensive search across several electronic databases, including PubMed, Embase, Scopus, and the Cochrane Library. The search was based on keywords including “Mobile-bearing”, “Medial-pivot”, “Total knee arthroplasty”, “Total knee replacement” or “TKA” covering all relevant publications up to November 2024. Any disagreements were resolved by consulting the third author.

### Inclusion and exclusion criteria

This meta-analysis reviewed original articles involving patients undergoing primary TKA with either MB or MP prostheses. The analysis compared the two groups regarding complication rates, long-term functional outcomes, and patient satisfaction. Notably, the complications considered included infection, prosthesis loosening, revision surgery, and limitation of movement. We included studies having reports of clinician-reported outcome measures, such as the Knee Society Score (KSS) and knee range of motion (ROM), and patient-reported outcome measures such as the Western Ontario and McMaster Universities Arthritis Index (WOMAC). Excluded from the screening were case reports, case series, expert opinions, reviews, letters, and systematic reviews. Studies involving patients who had previous knee surgeries, such as tibial plateau, distal femur, or patellar fixation, were also omitted. Additionally, studies without full texts or essential data were excluded, with no limitations on language or publication year. Any disagreements raised by the reviewers were addressed and resolved by the third author to reach a final conclusion.

### Data extraction and methodological quality

Two investigators independently assessed potentially relevant studies and extracted data from the articles selected for inclusion. The data collected encompassed the publication year, country of origin, research design, complication rates, long-term functional outcomes, patient satisfaction, and follow-up duration. The quality of nonrandomized controlled trial (non-RCT) studies was evaluated using the Newcastle–Ottawa scale [19], which assigns ratings from 0 to 9, with higher scores reflecting better quality and reduced risk of bias. The reviewers assessed each cohort or case–control study on the basis of three criteria: group selection, group comparability, and the ascertainment of outcomes or exposures, utilizing eight specific questions for this assessment. The quality of randomized controlled trials (RCTs) was assessed using the Cochrane Collaboration’s Risk of Bias Tool 2 (ROB2) [20], categorizing risk as low, high, or unclear across five domains: randomization process, deviations from intended interventions, missing outcomes, outcome measurement, and selection of reported results. Any disagreements were resolved by consulting a third reviewer for a final determination.

### Outcomes of interest

The main outcome of the study focused on functional outcomes, specifically the Knee Society Score, ROM, and WOMAC. Another outcome included patient satisfaction, which was observed at the final follow-up.

### Statistical analysis

This meta-analysis was performed using Review Manager software (version 5.4; Cochrane Collaboration, Oxford, UK). Continuous variables were reported as means with standard deviations. Data from each study were combined for analysis. For dichotomous outcomes, summary statistics were expressed as odds ratios (ORs) with 95% confidence intervals (CIs), calculated using the Mantel–Haenszel method. Continuous outcomes were presented as mean differences with 95% CIs using the inverse variance method. Heterogeneity among studies was evaluated with the *C*^2^ test, where significance was set at a *p* value below 0.1. The *I*^2^ test was also applied: values below 25% indicated low heterogeneity, 25–50% indicated moderate heterogeneity, and values above 50% indicated high heterogeneity. A random effects model was utilized when significant heterogeneity was present (*I*^2^ > 50% and *p* < 0.05), while a fixed effects model was applied otherwise. The results were illustrated using forest plots. Additionally, sensitivity analyses were conducted by removing one study at a time if significant heterogeneity was detected. Statistical significance was defined as a *p*-value < 0.05.

## Results

### Literature search

**Fig. 1:**
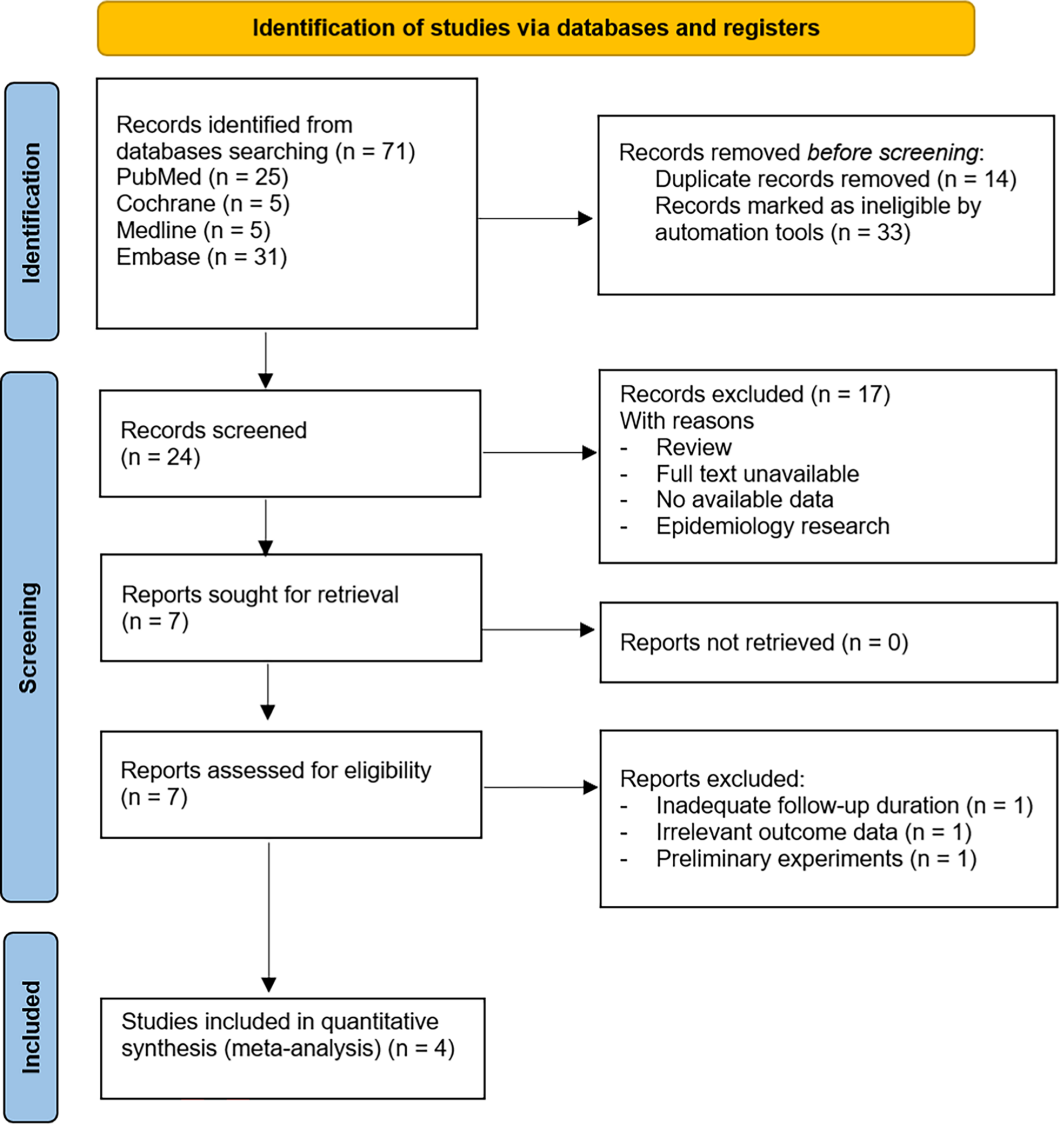
The 2020 PRISMA flow diagram of included studies

Figure 1 illustrates a flowchart of the study screening and selection process. A total of 40 studies were collected and screened from the electronic databases without language restriction. After removing 14 duplicating research and 33 ineligible studies, the remaining 24 documents remained. A total of 17 studies were deemed irrelevant and unavailable, leaving 7 studies to be assessed for eligibility. After excluding three studies owing to unavailable outcome data, the agreement was obtained on four studies for final inclusion after full-text review. As a result, three retrospective cohort studies and one RCT study remained to perform the systematic review and meta-analysis.

### Study characteristics

Table 1 presents the overview characteristics of the included studies. The four studies were conducted between 2016 and 2021 in Korea [5, 14], France [7], and India [18], with a total of 1069 patients and a pooled mean age of 68.21 years. These studies comprised three retrospective cohort studies [5, 7, 18] and one prospective RCT [14] in 1251 knees, which are replaced by the technique of TKA in the design of MB (632 prostheses) and MP (619 prostheses). All the studies reported long-term follow-up, which ranged from 5 to 11 years.

**Table 1:** The overview characteristics of included studies

No	Study (year)	Country	Design	Sample size (1069 patients)		Number of knees replaced (1251 knees)			Mean age (years) 68.21	Follow-up
				Total	Male (%)	Mobile-bearing	Medial-pivot	Total		
1	Choi (2016)	Korea	Retrospective cohort study	101	89 (88.12)	52	49	101	67.07 ± 7.13	5 years 64 months (60–72)
2	Jenny (2020)	France	Retrospective cohort study	670	254 (37.91)	334	336	670	69.67 ± 7.34	At least 10 years
3	Kim (2017)	Korea	Randomized controlled trial	182	52 (28.57)	182	182	364	65.60 ± 6.90	11 years (11–12.6)
4	Shakya (2021)	India	Retrospective cohort study	116	50 (43.10)	64	52	116	66.49 ± 5.86	7.1 ± 1.1 (years)

### Study quality

Supplementary Table 1 presents the methodological quality of all included studies. The prospective RCT included was evaluated by the Cochrane Collaboration’s Risk of Bias Tool 2, with most aspects evaluated as having a low risk of bias. The remaining three cohort studies were assessed by the Newcastle–Ottawa scale, which showed that two studies have 9 points and the other has 8 points. In general, three cohort studies were considered to have low risk of bias. In addition, as the number of studies was lower than ten studies, we did not apply the funnel plot to assess publication bias in this meta-analysis.

### Knee Society Score

**Fig. 2:**
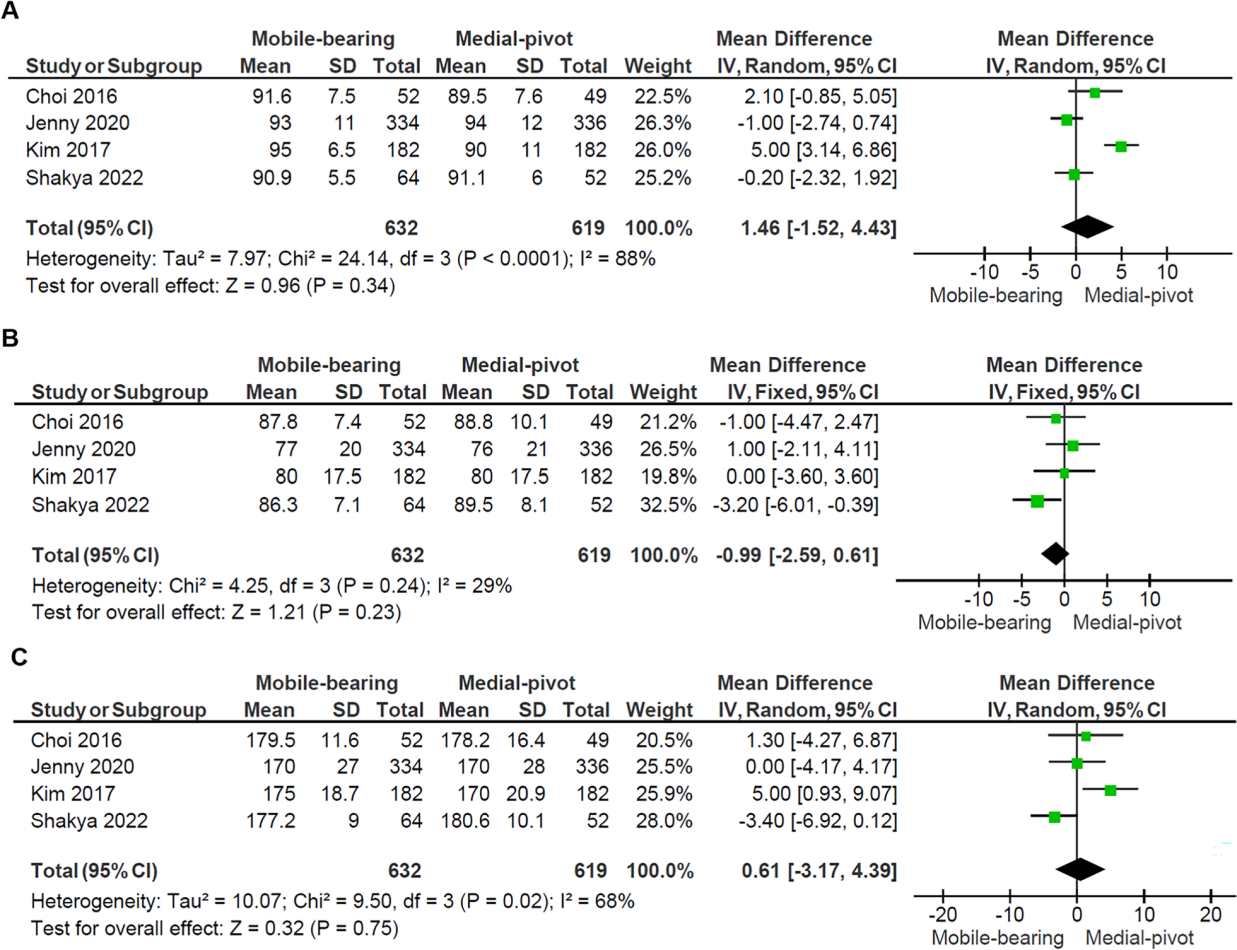
Forest plots comparing the **A** Knee Score, **B** Knee Function Score, and **C** Total Knee Society Score (KSS) between the mobile-bearing and medial-pivot design. *IV* inverse variance, *CI* confidence interval, *df* degree of freedom

Figure 2 details the forest plot of the KSS scores from the four studies [5, 7, 14, 18], including 1069 patients with 632 mobile-bearing knees replaced and 619 medial-pivot knees replaced. The domain Knee Score showed no significant difference between the two groups (*p* = 0.34), with a notably high heterogeneity among the studies (*I*^2^: 88%, *p* < 0.001). The domain Knee Function Score showed no significant difference between the two groups (*p* = 0.23), and no heterogeneity was observed between the studies (*I*^2^: 29%, *p* = 0.24). Subsequently, there was no significant difference in total KSS between the two groups (*p* = 0.75), with substantial heterogeneity detected among the studies (*I*^2^: 68%, *p* = 0.02).

### Knee range of motion

**Fig. 3:**
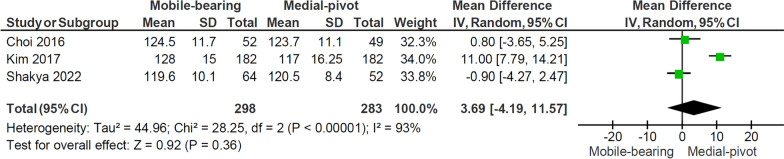
Forest plot comparing knee ROM at last follow-up between the mobile-bearing and medial-pivot design. *IV* inverse variance, *CI* confidence interval, *df* degree of freedom

Figure 3 shows the forest plot of the comparison of knee ROM between the two designs. Three studies [5, 14, 18]were included, comprising 399 patients with 298 MB knees replaced and 283 MP knees replaced. The meta-analysis showed no significant difference in knee ROM between the two groups (*p* = 0.36), with notable heterogeneity observed among the studies (*I*^2^: 93%, *p* < 0.001).

### The Western Ontario and McMaster Universities Arthritis Index

**Fig. 4:**
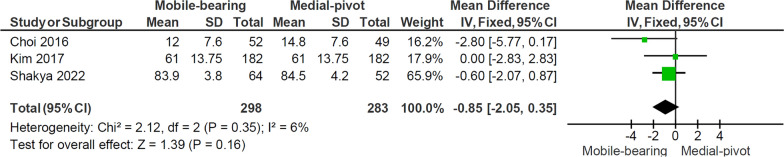
Forest plot comparing WOMAC score between the mobile-bearing and medial-pivot design. *IV* inverse variance, *CI* confidence interval, *df* degree of freedom

Three studies [5, 14, 18], including 399 patients with 298 MB knees replaced and 283 MP knees replaced, reported the WOMAC score. Figure 4 shows the pooled mean difference of the WOMAC score. There was no significant difference in the score between the two groups (*p* = 0.16), with no heterogeneity observed between the studies (*I*^2^: 6%, *p* = 0.35).

### Patient satisfaction

**Fig. 5:**
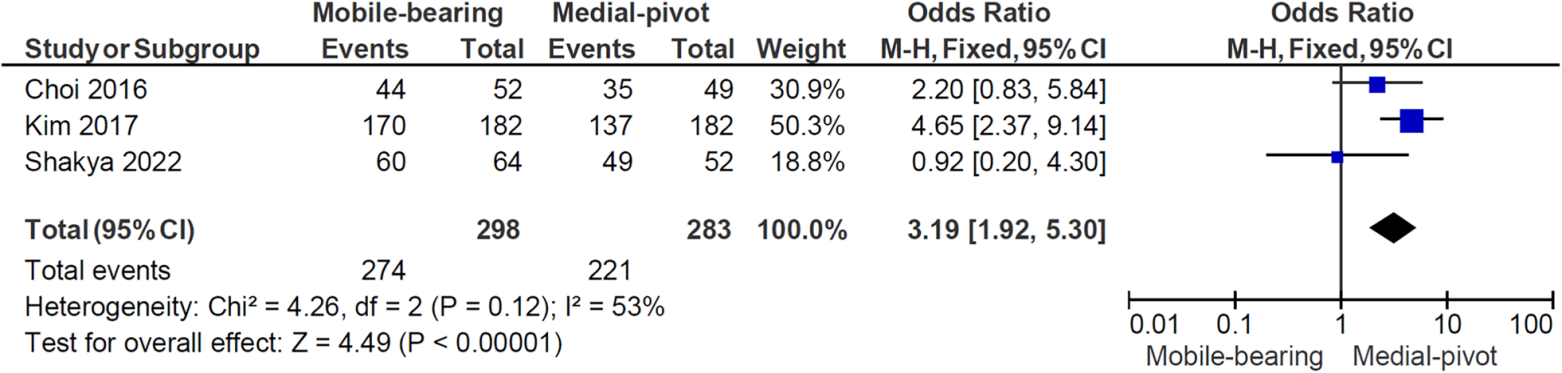
Forest plot comparing patient satisfaction between the mobile-bearing and medial-pivot design. *M–H* Mantel–Haenszel, *CI* confidence interval, *df* degree of freedom

Fig. 5 illustrates the forest plot of patient satisfaction from three studies [5, 14, 18], including 399 patients with 298 MB knees replaced and 283 MP knees replaced. The forest plot shows that patients with MB implants experienced more satisfaction than those with MP ones (OR 3.19, 95% CI 1.92–5.30, *p* < 0.001). No significant heterogeneity was observed between the studies (*I*^2^: 53%, *p* = 0.12).

### Complications

Supplementary Table 2 presents some common complications between the MB and MP groups after surgery. Three studies [5, 7, 18], including 887 patients with 450 MB knees replaced and 437 MP knees replaced, demonstrated the complications relating to revision surgery through radiolucent line and loosening. However, no significant difference was observed between the two groups. Additionally, Kim’s study, which included 182 patients with 182 knee replacements per group, reported significantly higher rates of ROM-related restriction and effusion in the MP group compared with the MB group [14].

### Sensitivity analysis

We conducted a leave–one–out sensitivity analysis for KS, KSS, and ROM outcomes. (Supplementary Figs. 1–3). Although there were notable reductions in heterogeneity among the studies, the results remained unchanged. This consistency underscores no statistically significant difference in functional outcomes between MB and MP prostheses.

## Discussion

This meta-analysis, incorporating data from 1069 patients and 1251 operated knees, offers insights into the comparative outcomes of MB and MP prostheses in TKA. While patient satisfaction emerged as a significant differentiator favoring MB prostheses, functional outcomes, as assessed using the KSS, WOMAC scores, and knee ROM, were similar across both designs.

### Knee Society Score (KSS)

The KSS is widely used for evaluating knee function and alignment post-TKA. Although the KSS is considered to have some limitations in assessing the kinematic TKA due to the current ambiguity in the “alignment” subcomponent, it has been a widely accepted assessment tool for providing functional evaluation in patients undergoing TKA. It incorporates both objective assessments of knee stability, ROM, and subjective patient feedback, such as pain and functional capacity [21, 22]. This meta-analysis revealed no significant differences in KSS outcomes between MB and MP prostheses, with scores exceeding 90 points in both groups in most studies, indicative of excellent functional performance. The lack of statistical significance across the Knee Score (*p* = 0.34), Knee Function Score (*p* = 0.23), and total KSS (*p* = 0.75) indicates that both implants provide comparable improvements in patient outcomes. However, the substantial heterogeneity observed in the Knee Score (*I*^2^ = 88%, *p* < 0.001) and total KSS (*I*^2^ = 68%, *p* = 0.02) suggests considerable variability among the included studies. This may stem from differences in patient demographics, surgical techniques, rehabilitation protocols, or study methodologies. This aligns with Wang Deng et al.’s validation of KSS as a reliable indicator for knee function, even in advanced arthroplasty procedures [23]. Additionally, prior studies compared the MB and posterior-stabilized design with no significant difference in the KSS domain after 10-year follow-up [9], others also reported similar KSS between the posterior-stabilized and MP in the Chinese population with satisfactory midterm outcomes [24]. Despite the indirect comparison, the similar concept underscores that both MB and MP designs are capable of achieving high levels of functional restoration after TKA.

### Knee range of motion (ROM)

ROM is a pivotal parameter for postoperative recovery, influencing activities of daily living and patient satisfaction. In fact, three out of four studies have been conducted with no significant differences in ROM between MB and MP designs. Both prostheses demonstrated substantial improvements in ROM from preoperative baselines, maintaining recovery trajectories over follow-up periods extending up to 11 years. The MP design enhances stability by minimizing condylar translation and replicating native knee kinematics, while the MB design promotes sagittal kinematics and deep flexion through a congruent articular surface [10, 25–27]. Kim et al. and Shakya et al. found no significant differences in femoral offset changes between MP and MB designs pre- and postsurgery, with both showing functional improvement [14, 18]. However, the studies did not assess variations in lateral condyle displacement during knee movement. Other studies demonstrated that patients with medial pivot implants experienced limited implant displacement during overground activities involving knee flexion, which can enhance the stability of the medial compartment at mid-flexion angles during weight-bearing activities and reduce condylar translation compared with native knees [1, 18, 28]. Otherwise, despite difference in kinematic feature with MP design, the MB design is associated with potential benefits for recreating more physiological gait mechanics by providing a congruent articular surface and reducing contact stresses [2, 27].

### WOMAC score

The WOMAC score evaluates pain, stiffness, and physical function in patients with osteoarthritis and is a critical tool for gauging outcomes after TKA [9, 14, 29]. Studies included in this meta-analysis reported comparable WOMAC scores between MB and MP groups over extended follow-up periods. This suggests that both implants offer comparable levels of pain relief, stiffness reduction, and functional improvement from the patient’s perspective. Notably, the low heterogeneity (*I*^2^ = 6%, *p* = 0.35) suggests strong consistency in the results across studies, implying that variations in study populations, surgical techniques, and rehabilitation protocols had minimal impact on WOMAC outcomes [29, 30]. While Choi et al. [5] and Shakya et al. [18] suggested better WOMAC scores for MB implants, long-term evaluation indicates insufficient evidence to conclusively demonstrate that MB implants result in superior WOMAC outcomes compared with MP implants. As such, the aggregated evidence remains insufficient to favor either design conclusively. The similar WOMAC outcomes suggest that both MB and MP prostheses are effective in addressing the symptoms and functional impairments of advanced knee osteoarthritis. Further research is needed to clarify the impact of these variables and to provide a more comprehensive understanding of functional outcomes following surgery.

### Patient satisfaction

Patient satisfaction, often regarded as the ultimate outcome in TKA, is influenced by multifaceted factors beyond traditional clinical metrics. Despite the generally favorable outcomes following TKA, studies estimate that 15–25% of patients remain dissatisfied after surgery [31, 32]. A key finding in our study was the notable difference in patient satisfaction between the two groups. The increased satisfaction with the MB design may be attributed to its ability to more closely replicate natural knee kinematics, offering enhanced ROM, adaptability, and a smoother, more natural feel during daily activities. Several factors may explain this higher satisfaction with MB implants. One key advantage of MB designs is their ability to better replicate natural knee kinematics by allowing rotational movement, which can improve joint flexibility, enhance proprioception, and reduce implant-related discomfort. This increased mobility may translate into smoother motion during daily activities, such as walking, squatting, and climbing stairs, leading to a more natural knee feel and better functional adaptation [10, 29]. Additionally, the ability of MB implants to distribute loads more evenly may help minimize polyethylene wear, potentially reducing pain and swelling, which aligns with patient expectations for comfort and implant longevity [33, 34]. In contrast, the MP design, while designed to provide greater stability through a more constrained motion pattern, may feel more restrictive, especially for active individuals who frequently engage in deep knee flexion [6, 14, 31]. This could explain why some patients perceive MP designs as less comfortable or adaptable, leading to lower satisfaction scores. The mismatch between patient expectations and implant performance is a crucial consideration, as dissatisfaction often arises when the perceived function does not align with preoperative expectations [14, 31, 35]. These differences highlight the importance of considering patient activity levels, expectations, and biomechanical needs when selecting TKA designs, as optimizing these factors can significantly influence postoperative satisfaction and outcomes.

### Complications

Complications following TKA with the two designs vary, particularly regarding infection and revision rates. Kim et al.’s study reported a higher incidence of infections and revision surgeries in patients with MP implants compared with MB implants, with infection rates for MP implants ranging from 0.2% to 0.4%. Infection remains the leading cause of revision surgeries in MP designs, often requiring interventions, such as intravenous antibiotics and antibiotic-loaded bone cement, which can extend hospital stays, increase costs, and delay recovery [4, 14, 17]. Additionally, MP implants have been associated with higher dissatisfaction and revision rates, which may partly be explained by instability owing to posterior cruciate ligament sacrifice and stress concentration at the implant-bone interface [16, 36]. Over time, these factors may contribute to aseptic loosening, a common cause of TKA failure [37]. While MP designs aim to replicate natural knee kinematics, the potential for instability and loosening highlights the need for further research to refine implant design and improve patient outcomes [5, 14, 18].

Besides, the MB platform also has some issues, such as complications of loosening, instability, and dislocation, which increase the risk of revision surgery [38, 39]. It differs from the MP design in that infection is not the main cause of revision; therefore, the complications appearing in MB platform TKA can result from component design. The possible reason for those complications is the movement of the tibial insert relative to the tibial tray in anterior–posterior translation and internal–external rotation, which causes loosening of the tibial component. Some kinematic studies showed that the insert could not follow the rapid movement of the femoral component, which consequently could result in a shift of the contact area with higher contact pressure [27, 40–42].

This meta-analysis has several limitations. First, the small number of included studies limits the statistical power and comprehensiveness of our findings. Second, only three out of four studies provided data on functional scores and patient satisfaction, which may reduce the robustness and reliability of the conclusions. Additionally, the predominance of studies conducted in Asian populations introduces potential regional or cultural biases, as the unique functional demands of this demographic, such as squatting and kneeling, may not be representative of other populations. These limitations underscore the need for more diverse, multicenter studies with larger sample sizes to enhance the generalizability and applicability of the results.

Despite these limitations, our study has several notable strengths. The extended follow-up period, spanning from at least 5 to 11 years, provides valuable insights into the durability and long-term outcomes of MB and MP prostheses. By integrating both objective and subjective assessment tools, our analysis offers a comprehensive evaluation of functional outcomes. These strengths, coupled with the acknowledged limitations, highlight the need for future research, particularly more RCTs comparing these two designs, in both short-term and long-term follow-ups, to further validate our findings and improve clinical practice in optimizing TKA outcomes.

## Conclusions

Our meta-analysis of four studies highlighted the long-term outcomes of TKA between MB and MP designs. While KSS, WOMAC scores, and knee ROM showed no significant differences, patients with MB designs reported higher satisfaction levels compared with those with MP designs.

## Supplementary Information

Supplementary Material 1

Supplementary Material 2

## Data Availability

All data analyzed in this study are included in these published articles.

## Abbreviations

MB: Mobile-bearing
MP: Medial-pivot
TKA: Total knee arthroplasty
ROM: Range of motion
WOMAC: Western Ontario and McMaster Universities
KSS: Knee Society Score
