# Supplementary material for: Mobile-bearing versus Medial-pivot Designs in Total Knee Arthroplasty: A Meta-analysis
Source: Knee Surg Relat Res. 2025 Jul 24;37:30. doi: 10.1186/s43019-025-00280-7 (PMC12291247; doi:10.1186/s43019-025-00280-7)
Supplement: Supplementary file 1 — Supplementary Material 1 [file 43019_2025_280_MOESM1_ESM.docx]

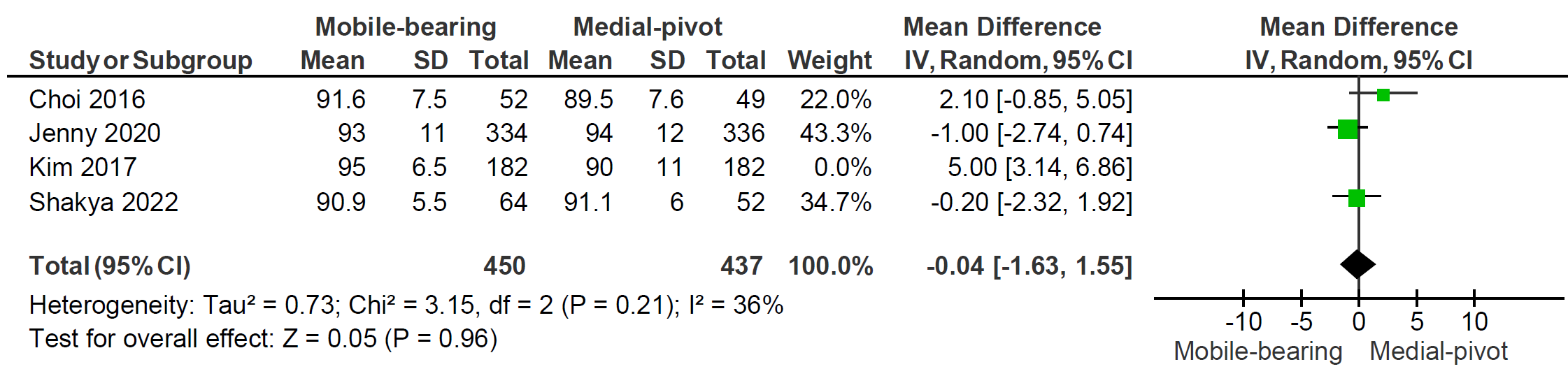


Supplementary Figure 1. Forest plot after sensitivity analysis of KS at the last follow-up. IV, inverse variance; CI, confidence interval; df, degree of freedom; KS, Knee Score


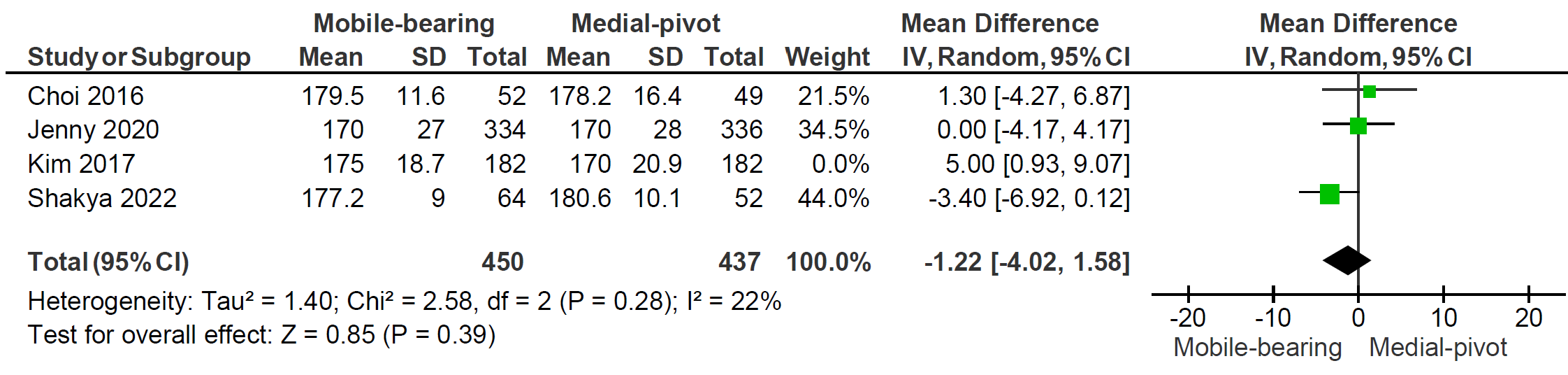


Supplementary Figure 2. Forest plot after sensitivity analysis of KSS at the last follow-up. IV, inverse variance; CI, confidence interval; df, degree of freedom; KSS, Knee Society Score.


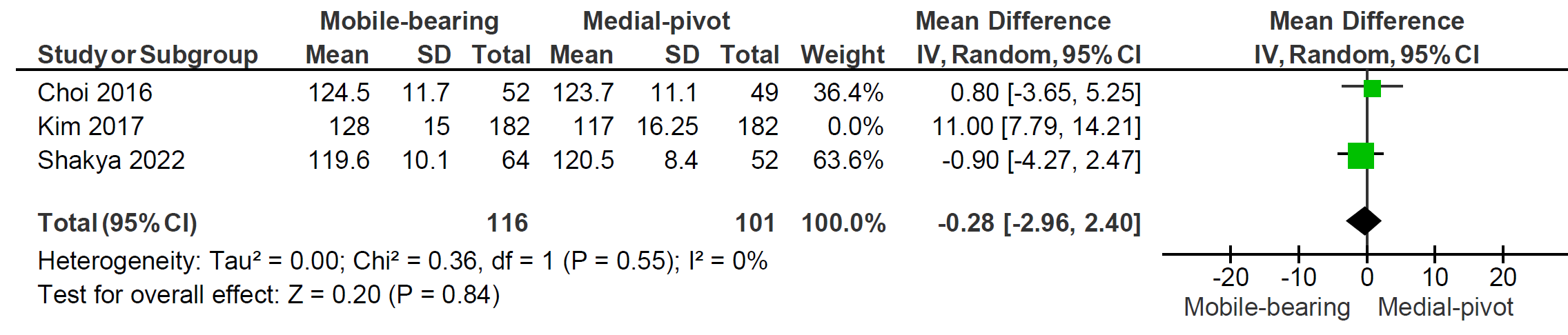


Supplementary Figure 3. Forest plot after sensitivity analysis of knee range of motion at the last follow-up. IV, inverse variance; CI, confidence interval; df, degree of freedom.
