# Supplementary material for: Mobile-bearing versus Medial-pivot Designs in Total Knee Arthroplasty: A Meta-analysis
Source: Knee Surg Relat Res. 2025 Jul 24;37:30. doi: 10.1186/s43019-025-00280-7 (PMC12291247; doi:10.1186/s43019-025-00280-7)
Supplement: Supplementary file 2 — Supplementary Material 2 [file 43019_2025_280_MOESM2_ESM.docx]

Supplementary Table 1

Risk of bias assessment

| **No** | **Study (Year)** | **Design** | **Risk of Bias Assessment According to the Newcastle – Ottawa Scale** | | | | | | | | |
| --- | --- | --- | --- | --- | --- | --- | --- | --- | --- | --- | --- |
|  |  |  | **Selection** | | **Comparability** | | **Exposure/Outcome** | | | **Total** | |
| 1 | Choi (2016) | Retrospective cohort | **** | | * | | *** | | | 8 | |
| 2 | Jenny (2020) | Retrospective cohort | **** | | ** | | *** | | | 9 | |
| 3 | Shakya (2022) | Retrospective cohort | **** | | ** | | *** | | | 9 | |
| **No** | **Study (Year)** | **Design** | **Risk of Bias Assessment According to the Newcastle – Ottawa Scale** | | | | | | | | |
|  |  |  | **Domain 1** | **Domain 2** | | **Domain 3** | | **Domain 4** | **Domain 5** | | **Overall** |
| 1 | Kim (2017) | Prospective randomized control trials | Low | Low | | Low | | Low | Low | | Low |

Supplementary Table 2

Complications of MP and MB designs in the included studies

| **No** | **Study (Year)** | **Loosening and instability-related sign** | **ROM-related restriction** |
| --- | --- | --- | --- |
| 1 | Choi (2016) | 7.7% MB and 6.1% MP: Radioculent line  (p = 0.889) | NA |
| 2 | Jenny (2020) | 12/334 MB and 10/336 MP: Revision  (p = 0.67) | NA |
| 3 | Kim (2017) | 9% MP and 1% MB: Effusion  (p = 0.04) | 4% MP and 0.5% MB: pain and stiffness  (p = 0.05)  10% MP and 4% MB: Insufficient ROM  (p = 0.02)  3% MP and 0.5% MB: Infection  (p = 0.05) |
| 4 | Shakya (2022) | 2/52 MP and 3/64 MB: Radioculent line | 1/52 MP and 1/64MB: Wound gaping  3/52MP and 3/64MB: Flexion deformity |

Abbreviations: MP – Medial-pivot, MB – Mobile-bearing, NA – Not available
